# Implementing a Complex Intervention to Support Personal Recovery: A Qualitative Study Nested within a Cluster Randomised Controlled Trial

**DOI:** 10.1371/journal.pone.0097091

**Published:** 2014-05-29

**Authors:** Mary Leamy, Eleanor Clarke, Clair Le Boutillier, Victoria Bird, Monika Janosik, Kai Sabas, Genevieve Riley, Julie Williams, Mike Slade

**Affiliations:** 1 King's College London, Institute of Psychiatry, Denmark Hill, London, United Kingdom; 2 2gether NHS Foundation Trust, Rikenel, Montpellier, Gloucester, United Kingdom; Maastricht University Medical Centre, Netherlands

## Abstract

**Objective:**

To investigate staff and trainer perspectives on the barriers and facilitators to implementing a complex intervention to help staff support the recovery of service users with a primary diagnosis of psychosis in community mental health teams.

**Design:**

Process evaluation nested within a cluster randomised controlled trial (RCT).

**Participants:**

28 interviews with mental health care staff, 3 interviews with trainers, 4 focus groups with intervention teams and 28 written trainer reports.

**Setting:**

14 community-based mental health teams in two UK sites (one urban, one semi-rural) who received the intervention.

**Results:**

The factors influencing the implementation of the intervention can be organised under two over-arching themes: *Organisational readiness for change* and *Training effectiveness*. Organisational readiness for change comprised three sub-themes: NHS Trust readiness; Team readiness; and Practitioner readiness. Training effectiveness comprised three sub-themes: Engagement strategies; Delivery style and Modelling recovery principles.

**Conclusions:**

Three findings can inform future implementation and evaluation of complex interventions. First, the underlying intervention model predicted that three areas would be important for changing practice: staff skill development; intention to implement; and actual implementation behaviour. This study highlighted the importance of targeting the transition from practitioners' intent to implement to actual implementation behaviour, using experiential learning and target setting. Second, practitioners make inferences about organisational commitment by observing the allocation of resources, Knowledge Performance Indicators and service evaluation outcome measures. These need to be aligned with recovery values, principles and practice. Finally, we recommend the use of organisational readiness tools as an inclusion criteria for selecting *both* organisations *and* teams in cluster RCTs. We believe this would maximise the likelihood of adequate implementation and hence reduce waste in research expenditure.

**Trial Registration:**

Controlled-Trials.com ISRCTN02507940

## Introduction

In England, current mental health policy states that ‘More people with mental health problems will recover’ [1]. Used in this context, recovery refers to processes which enable individuals to live a fulfilling, hopeful and contributing life, with or without symptoms of illness [2]. Whilst not unusual, there is still a translation gap between this ‘adoption in principle’ of recovery-oriented practice at a national policy level and local mental health practice [3]. Organisational transformation has been identified as one of three scientific challenges for implementing recovery practices internationally [4]. There is now a growing body of literature offering recovery-practice guidance. For instance, Farkas and colleagues set out examples to show how recovery-orientated mental health programmes need to ensure both organisational/administrative and staffing dimensions are underpinned by values-based recovery standards (person orientation, person involvement, self-determination/choice, growth potential) [5]. Similarly, Davidson and colleagues have also given practical advice for those who can influence system-level changes [6].

The evidence base on factors which lead from the ‘adoption in principle’ to the ‘early implementation’ and ‘persistence of implementation’ phases of embedding a complex recovery intervention within routine mental health practice is currently limited. In particular, there is a knowledge gap around staff perspectives on the barriers and facilitators to implementing a recovery intervention in mental health services. Whitley and colleagues investigated factors which affected implementation of a combined recovery and illness self-management intervention in community mental health centres in the United States [7]. They found that at the organisational level of behaviour change, four factors determined the success or failure of implementation of the illness self-management and recovery programme: leadership, organisational culture, training and staff supervision. Implementation studies have previously contributed to changing practice within psychiatry, for instance, the recent update of the NICE guidelines for psychosis and schizophrenia in adults [8] was informed by studies looking at barriers to implementation of psychological interventions and mental health guidelines [9] [10].

The REFOCUS intervention is a complex intervention to support recovery [11] which is being evaluated within a cluster RCT in South London and Maudsley (SLaM) NHS trust and 2gether NHS trust, in Gloucestershire (see Method). Complex interventions involve a number of components, each of which may act both independently and inter-dependently [12] and target multiple behaviours at different levels of healthcare systems. They are especially prone to problems around design, implementation and evaluation [13]. In a BMJ editorial, Thompson highlighted the need for evidence not only on whether interventions are effective and should be implemented, but also on what can generally aid efforts to implement complex interventions more widely [14].

Following guidance from the latest Medical Research Council (MRC) framework for developing and evaluating complex interventions [15], a process evaluation was conducted in parallel with the main RCT. This present study, which formed part of the process evaluation, aimed to identify wider contextual and individual factors which promote or inhibit efforts to implement complex interventions into existing mental healthcare practice.

The study was approved by East London Research Ethics Committee (Ref. 11/LO/0083) on 22/2/11.

## Method

A cluster randomised controlled trial was used to evaluate the effectiveness of the REFOCUS intervention at increasing staff support for personal recovery. The 12 month, team-level intervention was delivered to healthcare professionals who all provide care co-ordination (Recovery, Psychosis and Forensic teams). The intervention was designed to change mental health care practice from the bottom-up, i.e. at both a practitioner and team level, rather than from a top-down, organisational level. 14 community-based mental health teams were allocated to the intervention arm and entered the trial in separate geographically-based waves via block randomisation between April 2011 and May 2012. All teams completed the intervention by September 2013. All staff that provided mental health care within a team were included, regardless of discipline, qualifications, or experience. Within the trial, outcome data was only collected from people with a primary diagnosis of psychosis. As the intervention was provided to teams whose caseload was broader than this, some recipients of the intervention had other mental health diagnoses.

The REFOCUS intervention was theory-based [16], and targeted two of four dimensions identified from an international review of best practice in supporting recovery, namely ‘working relationship’ and ‘supporting personally defined recovery’ [17]. First, recovery-promoting relationships were supported by training teams to use coaching skills in their clinical interactions, and facilitating ‘Partnership Projects’ involving staff and service users to undertake a joint activity outside of formal roles. Second, support for personally-defined recovery was addressed by training and supporting staff behaviour change in relation to three working practices: understanding values and treatment preferences, assessing and amplifying strengths, and supporting goal-striving by the service user. A testable REFOCUS Model identifying active ingredients and causal pathways between intervention and outcome was published [16]. Six implementation strategies were used: separate information sessions for staff and service users; personal recovery training (10.5 hours); coaching and working practice training (14.5 hours); team manager reflection sessions focussed on team culture (3 hours externally facilitated by the Personal Recovery trainer) and whole team reflection sessions (3 hours externally facilitated, 3 hours internally facilitated by team) focussed on reinforcing behaviour change and individual supervision focussed on reflective practice development. The REFOCUS manual and training materials are all freely available to download at researchintorecovery.com/refocus. A summary table of the intervention and implementation strategies are provided in tables 1 and 2.

**Table 1:** Summary of REFOCUS intervention components.

**Component 1: Recovery-promoting relationships**
Developing a shared team understanding of personal recovery
Exploring individual and team values
Skills training in coaching
Teams carrying out partnership project with service users
Raising the expectations held by service users that their values, strengths and goals will be prioritised
**Component 2: Working practices**
Values and treatment preferences
Strengths
Personally- valued goals

**Table 2:** Summary of REFOCUS implementation strategies.

Implementation strategy	To whom	Length of time	Month
Information sessions for staff and service users	Provided to team	1 hour	Month 1
Personal recovery training	Provided to team	3× half days	Month 1, 2 and 5
Coaching conversations for Recovery training	Provided to team	1 full and 2 half days	Month 3, 4, 5
Team reflection sessions	3 externally facilitated	1 hour	Month 2, 4, 10
Team leader reflection sessions	6 externally facilitated	1 hour	Month 1, 3, 6, 9, 12
Individual Supervision	Self-organised by team	Part of clinical supervision	Ongoing

This research took place at a time of national policy changes to mental health care services, such as *public sector targets for significant cost-savings*, leading to pressures on organisations to re-evaluate their priorities, streamline and reconfigure their services. Additionally, a new financing system, *Payment by Results* to make payments contingent on independently verified results lead to new organisational initatives and targets, along with the introduction of *Direct Payments* from social services to service users, enabling them to buy care services for themselves.

Significant unforeseen organisational changes occurred since the study planning stages which impacted upon the ability of teams to participate in the trial and implement the intervention. In SLaM NHS trust, clinical services were previously configured according to geographical location, with services being provided at a borough level. Before and during the trial, services and care pathways were reorganised around psychiatric diagnosis, creating *Clinical Academic Groups* (CAG), as part of the preparation for the possible merger of three NHS foundation trusts with King's College London, to form a single academic health centre called King's Health Partners. Other organisational initiatives included the introduction of SLaM recovery care plans, requiring these to be written in the first person. The 2gether NHS trust introduced a local non-discriminatory mental health service model called ‘*Fair Horizons*’. This led to existing teams being merged into ‘one stop teams’, giving a single access point for all working age adult, older age adult, child and learning disability referrals.

### Participants and methods

#### Individual interviews

28 face to face, in-depth interviews were conducted with staff and team leaders from intervention teams. A purposive sample with maximum variation (for profession, gender, experience in mental health services, team, intervention wave) were approached to participate. The two inclusion criteria were i) working clinically in a REFOCUS intervention team, and ii) self-reported use of the intervention. Recruitment continued until category saturation was reached. Individual interviews were conducted at mid-point (n = 4) and end-point (n = 24) between December 2011 and August 2013. In addition, three mid-point interviews were conducted with trainers to explore their experiences of delivering the training and working with individual teams.

#### Focus groups

We recruited a purposive sample of four intervention teams which varied across site and wave (n = 24 participants). As recommended by Morgan [18], we invited between six to eight staff to participate in the focus group to represent the range of views within the team. These end-point focus groups were held at community mental health team bases between January 2013 and July 2013.

#### Trainer reports

Separate Personal Recovery (n = 14) and Coaching for Recovery (n = 14) Training reports were prepared for each intervention team.

### Setting and recruitment

Interview and focus group participants were recruited from the two trial sites either face-to-face or via telephone. The four South London boroughs are urban, with high levels of socio-economic deprivation and 55% of population come from white or white minority backgrounds [19]. In contrast, Gloucestershire is predominantly rural, with lower levels of socio-economic deprivation, and 95% of the population come from white British or white minority backgrounds [19]. The majority of interviews and all focus groups were held at community team bases. All participants were provided with an information sheet which outlined the purpose of the study, given an opportunity to ask questions and asked to sign a consent form. Of the potential interviewees approached, one person refused to be interviewed because they were too busy, whilst another expressed an interest in being interviewed and then was uncontactable. One team initially agreed to participate in a focus group, but then changed their minds when they became aware their team was being disbanded.

### Data collection and sampling

Focus groups were co-facilitated with a lead facilitator taking on the role of asking questions and managing group processes and a second facilitator taking notes, observing and asking supplementary questions (GR & ML in 2gether; ML & EC in SLaM). Groups lasted between 60–90 minutes.

Face to face interviews with staff and trainers were conducted by six interviewers (EC, ML, CLB, MJ, VB and GR). All interviewers were closely supervised by senior researchers, regularly met to discuss the development of the interview schedules and focus group topic guides, observed others, did role play interviews, to ensure there was a shared understanding of how to use interview schedules in practice. In six interviews a junior and senior researcher was present for training purposes. All interviews lasted between 45–60 minutes.

### Materials

#### Training reports

Trainers provided two-page written reports on the six intended practice change areas of team values, individual values, knowledge, skills, behavioural intent and behaviour, set out in the REFOCUS intervention model. Sample questions included ‘What were your impressions of the training overall?’ ‘What worked well and what didn't work well?’ ‘How well was the training received?’ ‘Were there differences between professional groups?’ (See Figure S1 Training report guide).

#### Interview schedule

The interview guide for staff and team leaders was developed in consultation with our Lived Experience Advisory Panel (LEAP) of service users and carers and piloted in the mid-point interviews. It was subsequently revised, with additional questions and prompts being added for each of the intervention components. The final version of the staff interview schedule covered perspectives on the whole intervention, its components, and factors which influenced the feasibility and implementation of the intervention. Sample questions included ‘How has the coaching training altered how you work with service users?’ ‘Can you give an example in the last 6 months of when you have assessed a clients strengths?’ ‘Has the REFOCUS intervention changed your relationships with clients and if so, how?’ ‘How has reflection supported you to implement the REFOCUS intervention?’ (See Figure S2 Staff Interview Schedule).

#### Focus group topic guide

The focus group topic guide covered participant's understanding of recovery, experiences of delivering the intervention and views on what had contributed to their success or failure at implementing the intervention. Sample questions included ‘As a team, how have you found implementing the REFOCUS Manual with your service users?’ ‘What is it about your team that enables you to successfully support recovery?’ ‘What has helped or hindered your team in implementing the intervention?’ (See Figure S3 Focus group topic guide).

### Data analysis

We followed Braun and Clarke's six-phase guide for inductive thematic analysis and used the qualitative data analysis package NVivo (version 9) [20]. We digitally recorded interviews and focus groups, transcribed recordings verbatim, checked, anonymised and re-read them to increase familiarisation. At participant's request, two transcripts were returned for checking, but no corrections or comments were recieved. Particular attention was paid to any deviant cases as we were keen to compare and contrast the reasons why practitioners or teams had been especially successful or hindered in their attempts to implement the intervention.

Firstly, a sample of the interviews and trainers' reports were analysed jointly (ML, MJ, EC) to create a list of initial codes, which were then merged, refined and sorted into a hierarchy of more abstract, over-arching and sub-themes. Coders met to review their coded passages and to agree on the major themes, deviant cases and to discuss coding differences to arrive at a consensus. This process of investigator corroboration is designed to maximise the validity and trustworthiness and to safeguard against bias within the analysis process [21]. The initial coding framework was then used to analyse all staff interview (EC, ML), trainer interview (CL and ML) and focus group transcripts (ML, EC) and written reports (ML, MJ, KS). Data analysis and collection occurred concurrently. Data collection ended when it was judged that data saturation for the majority of themes had been reached.

### Findings

Socio-demographic data on staff (n = 41) and team leader (n = 11) participants is shown in table 3.

**Table 3:** Characteristics of staff participants.

	Interviews (n = 28) Mean (SD)	Focus Groups (n = 24) Mean (SD)
**Age** (years)	46.76 (10.216)	44.19 (8.152)
**Time since Qualified** (months)	228.52 (121.139)	202.50 (110.534)
**Time in Mental Health Services** (months)	213.11 (110.228)	189.35 (92.723)
**Time in post** (months)	62.93 (59.623)	54.41 (40.060)
**Gender n(%)**		
Male	11 (39)	7 (29)
Female	17 (61)	17 (71)
**Ethnicity**		
White British/White Irish/White other	23 (82)	16(67)
Black/Black British-African/Black British-Caribbean/Black	2 (8)	6 (25)
Other Asian/Asian British-Other	1 (4)	1 (4)
Other	2 (7)	1 (4)
**NHS Trust**		
South London and Maudsley NHS Foundation Trust	19 (68)	11 (46)
2gether NHS Foundation Trust	9 (32)	13 (54)
**Job Role**		
Staff	23 (89)	18 (25)
Team Leader	5 (18)	6 (75)
**Team**		
Support and Recovery team	25 (89)	22 (79)
Forensic, high support team	2 (7)	0 (0)
Psychosis team	1 (4)	2 (7)
Low intensity team	1 (4)	0 (0)
**Profession**		
Psychiatrist	4 (14)	2 (8)
Nurse	14 (50)	12 (50)
Psychologist	2 (7)	1 (4)
Social Worker	2 (7)	4 (17)
Occupational Therapist	2 (7)	1 (4)
STR Worker/Support worker	3(14)	2 (4)
Associate Practitioner	1 (4)	2 (8)
Physio technician	0 (0)	1 (4)
**Highest Qualification**		
A-level or equivalent/NVQ level	1 (4)	2 (8)
Higher national certificate/Diploma	6 (21)	6 (26)
Bachelors degree	9 (32)	7 (30)
Postgraduate degree	7 (25)	2 (8)
Other relevant professional training	5 (18)	6 (26)
Missing	0 (0)	1 (4)
**Grade**		
Bands 3 and 4	3 (11)	4 (18)
Band 5 and 6	15 (55)	12 (56)
Band 7 and 8a	5 (19)	4 (19)
Missing	5 (15)	4 (19)

The hierarchy of barriers and facilitators to implementing the intervention were organised under two higher order categories: Organisational readiness for change and Effective Training. The first higher order category, *Organisational readiness for change*, includes three sub-themes: i) NHS trust readiness, consisting of organisational commitment and organisational change, ii) Team readiness, consisting of effective leadership, team stability and composition and recovery practice baseline, and iii) Individual readiness, consisting of attitudes toward the trial and intervention, perceived fit with own existing values, knowledge or practices and willingness to apply to practice. The second higher order category: *Effective training*, includes three sub-themes: i) Engagement strategies and ii) Delivery style and content, iii) Modelling recovery principles. These are shown in table 4: Hierarchy of Themes.

**Table 4:** Hierarchy of themes.

Theme 1. Organisational readiness for change		
1.1 NHS readiness	1.2 Team readiness	1.3 Individual practitioner readiness
*1.1.1 Organisational change*	*1.2.1 Effective leadership*	*1.3.1 Attitudes about trial and recovery*
- Timing of intervention	- Attitude (opportunity or threat)	
-Job threats	-Leading by example	
-Increased task demand	-Containing leadership	
*1.1.2 Organisational commitment*	*1.2.2 Team stability and composition*	*1.3.2 Perceived fit with values, knowledge or practice*
- Organisational/commissioning priorities	- Stage of team development	
-Communication	-Team composition	
-Resource availability		
-Existing structures		
	*1.2.3 Recovery-practice baseline*	*1.3.3 Willingness to apply to practice*
	- Understandings of recovery	
	-Shared team approach to risk-taking	
	-Openness to critical reflection	
	-Presence of existing or would-be recovery champions	

Theme 2. Effective training		
2.1 Engagement strategies	2.2 Delivery style	2.3 Modelling recovery principles in training
*2.1.1 Validating existing skills*		
*2.1.2 Contracting*		
*2.1.3 Voluntary attendance*		

### Organisational readiness for change

#### NHS Trust readiness

Some clinicians were dubious about organisational commitment to supporting recovery practice. They felt that existing mental health services regard recovery as a peripheral, rather core purpose for mental health care.

*It* [Recovery-oriented practice] *needs to be priority, given a value within organisation, because it will otherwise get lost because managing risk, throughput, needing to do assessments will come first.The Trust needs to prove value for money. It needs space and time to allow individuals to be able to go over and beyond what the corporate measured expectations are, or find some sort of meaningful cost based outcome which someone is going to take seriously.* (Focus group 1, Participant 5, 2gether)

There were mixed views about whether senior managers had communicated the importance of the trial and intervention sufficiently. Some staff were angry that their Trust had continued supporting the trial at a time of considerable organisational change and financial cut-backs. Though the exact opposite view was also expressed:

*I'm not sure we've been influenced enough by our management to say actually this is really important, so you come in because there is this general grumbling about having something extra to do and you're influenced by that and actually you think, I've got more important things to do, which isn't right, it's just that's what the culture's like around you.* (Focus group 2, Participant 6, 2gether)

Participants felt that the wider organisation needed to visibly demonstrate their commitment to recovery practice through the provision of resources, both during and after the trial.

*In real world staff duties need to be covered. Future delivery of recovery training needs to accommodate these issues to enable teams to spend time together. We were getting behind on admin., reduction on team size led to more time on fire-fighting than recovery work, more time on those in crisis. We want the organisation to be more supportive. Recovery work isn't quick work.* (Focus group 3, Participant 2, SLaM)

Given the resource constraints, several clinicians felt that certain tasks within the intervention did not fall within their remit and should be carried out by care co-ordinators or support workers employed on lower pay grades.

*…so I can think of examples when I've tried to support the person with goal striving but there's interesting professional challenges that creates of you know, am I the right person? I am paid more than someone else, should I be sitting here getting paid to talk about how to make a friend?* (Interview, Participant 11, SLaM)

The organisational changes described above led to considerable re-structuring of services. This resulted in higher levels of staff turnover, workloads, staff stress and changes to team's skill mix, making teams unstable. The organisational changes were of such intensity that workers reported focussing upon and prioritising their own survival.

*I think to be fair, we felt nothing to do with the recovery project just what's been happening in the organisation has left us all feeling under siege and just fighting for our own mental health survival in a shrinking organisation.* (Focus group 2, Participant 3, 2gether)

Having to absorb more people onto caseloads, often ‘*at the heavy end*’ of the severity spectrum, led one worker to question whether the organisation should be focussing upon recovery and well-being agenda.

*Most people out in the world do not fulfil their potential. We might be able to make people feel less mentally unwell but I don't think we're going to get them to fulfil their potential. I think that is unrealistic, given the current state of the economy, resources and our time.* (Focus group 2, Participant 3, 2gether)

Participants reported a lack of time for reading the intervention manual, reflection, practicing new skills, using the individual recovery supervision guide and embedding the intervention into their existing practice with all their clients.

#### Team readiness

There were some clear differences between the teams in terms of leadership, stability, composition and their current level of recovery practice which affected their overall readiness.

Some team leaders and psychiatrists saw the REFOCUS intervention as an opportunity to establish the team's identity and creditials as a Recovery team, and/or provide the vehicle to enhance team-working. Others saw it as an extra burden, a threat to their professional identity, or resented it as an implied criticism of existing practice. At times, senior clinicians and team leaders actively blocked their team's efforts to become more recovery-focussed.

*They have a very dominant psychiatrist there and one thing they did express when s/he was absent, was that they will make collaborative care plans and they will have them blocked at that level. It will all just get wiped out and whatever they've planned if it's not what the psychiatrist wants, it's overruled.* (Trainer interview)

More positively, there were examples of team leaders, psychiatrists and other senior clinicians championing the intervention:

*The team leader, deputy and senior clinicians attended the sessions and I noted what a powerful message that conveyed to the rest of the team about the importance of the training and its application. The leadership was actively engaged and consequently we began from the position of ‘how to apply them’* [coaching model and 3 working practices] *rather than ‘whether we wish to accept them’.* (CfR training reports, Team 4)

Trainers listed the benefits of psychiatrists attending training as providing practical leadership in exercises and group discussions, endorsing the REFOCUS approach to supporting recovery, helping contextualise the learning, working with the trainer to ‘bring on the team’, offering robust enquiry, which all lead to greater attendance and fuller engagement of team.

*The Consultant Psychiatrist attended all sessions, demonstrated up front leadership, an elegant coaching style, whilst also modelling the acceptability of constructive challenge in a team setting. The input of this leadership has helped incredibly in making sure the programme translates from ‘just training’ to a ‘way of working with service-users’.* (CfR training report, Team 3)

There were differences between teams in the extent to which the leadership was sufficiently stable and able to lead the team during organisational changes.

Team readiness appeared to be linked to the life-cycle stage of team's development. Teams ranged from being newly formed, mid-life, relatively stable, mature teams though to ‘dying’ teams that were preparing either to merge with another team or be disbanded.

*I think the timing of the delivery was unfortunate and in a different phase of this team's ‘life-cycle’ would have been exceedingly well taken up and leveraged to best effect. As it was I was delivering training to a ‘dying team’ and while some valuable elements might still have embedded themselves in the team's practice, my guess is that this kind of training is a ‘development phase’ type of investment (provided ideally in the maturing/mid-life phase of a team's development where it has time to embed in practice effectively).* (CfR report, Team 13)

The trainers observed differences between teams in their values, beliefs, attitudes, knowledge and understandings of recovery. Some participants reported feeling that the Personal Recovery training did not acknowledge this and was pitched too low.

*I expected there to be some fairly consistent values or attitudes within teams within one area, or at least within the same Trust. I think what's been fairly staggering is how very different they. Basically [name of team] are so pro-recovery they see it in terms of personal autonomy, the need for a power shift, people leading their own treatment choices.* (Trainer interview).

The Personal Recovery training challenged participants to critically reflect upon areas of mental health care practice which may not always sit comfortably with recovery-practice, for example, having a duty of care, prescribing medication, risk-taking and the use of cohersion. Teams varied in the extent to which they felt willing and able to do this.

*In one of the scenarios put to them, someone who wanted to be discharged and hadn't been told they could be. This team were really shocked by that, but then thought hang on a minute, Do we really always do that? Do we ever let it be implied? And they really were prepared to investigate that and reflect on it and they don't feel threatened by uncovering something and this. The team manager really leads this, they don't feel at all threatened by this.* (Trainer interview)

*In the team now is openness because you can challenge colleagues, people won't take exception to you challenging them.* (Focus group 3, Participant 5, SLaM)

The training also revealed differences in what staff considered was coercive and acceptable, for instance;

*What the manager actually said was, ‘well it is true that I often say to people when they say they want to reduce their medication ‘oh for heaven's sake, don't come off your medication because you're well now and you'll relapse’ and she said ‘but surely that's not coercive?’ So I said ‘Well it's a kind of it's a strong word but it is a misuse of power, it is coercive. You're telling someone they will relapse, you're not saying to somebody ‘this is one of the possibilities let's look at the range of them and how could we help you with this’. The psychiatrist in that team, very honestly and openly said ‘yes we use coercion and we use it all the time and we can't pretend we don't.’* (Trainer interview)

Other differences between teams were around their attitudes and behaviour towards risk-taking. One team in particular seemed to have spent considerable time, prior to recovery training, discussing how to manage and share risk as a team, which held them in good stead when it came to discussing professional concerns around positive risk-taking within recovery-oriented practice.

*They don't just have a very good idea of what to do with risk, but they've clearly discussed that fully as a team, which I think has freed their thinking around recovery a bit, so they're not as risk-averse.* (Trainer interview)

In some teams, there were already individuals who were highly committed to implementing recovery practice not just within their own practice, but actively looking for ways of developing it within their whole team.

*The team leader, myself and the clinical leader came out of it saying ‘Brilliant, right now what are we going to do with this?’ because that's what we do each time, we come out and literally we will meet for half an hour an hour, usually instigate by me. Afterwards I've gone round to all different care coordinators and said, ‘That was really good, what would you like out of it? …because for us it's like as soon as it's happened that can be a catalyst for something else that we can set up in the team and embed it somewhere.* (Interview, Participant 3, SLaM)

#### Individual practitioner readiness

Individuals varied in their attitudes towards the trial and intervention, levels of recovery knowledge and belief in their own capabilities to integrate the intervention into their existing practice.

Some individuals expressed varying degrees of resentment and frustration that their team was required by their Trust to participate in the trial. At an individual level, people were able to refuse to consent to participate in the trial, but may still have felt undue pressure and the situation certainly created mixed messages.

*It went back to the fact that actually one had to question whether the everybody was truly consenting, because we were all subpoenaed into doing it, I mean we were told that this is what we would be doing, we weren't offered a choice.* (Interview, Participant 4, SLaM)

It was very common for clinicians to report that they felt they were already working in a recovery-oriented way and that the intervention did not offer them anything new.

*I'm one of those people who when we started with the study felt that honestly, we are doing what these people are saying, it's just that they are using a different name to actually give us more work to do, so that was my feeling ‘cos part of it we say okay is it really new information?* (Interview, Nurse, Participant 12, SLaM)

For some clinicians, the coaching skills and recovery training was highly consistent with their skills, values, attitudes and working style. They used the training to refresh their knowledge and skills.

*Coaching training, yeah, and I found that very, very beneficial. It's very similar to motivational interviewing and solution-focussed therapy but it was a good to go through these again.* (Interview, Nurse, Participant 13, SLaM)

*I do a lot of goal-setting but I think that's also because I have got a psychotherapy training, so I suppose I use those skills from there, but they were also refreshed.* (Interview, Participant 21, SLaM)

Some clinicians were able to quickly absorb the skills training and focus upon considering how and when to incorporate the approach into their routine practice;

… *the skills to notice that you have a choice as a clinician of using specifically more powerful or less powerful questions, just depending on what the presentation of the client is, and pitching it is stuff that wouldn't even be there in some of the other teams, but would have added a huge dimension to how effectively those staff coached.* (Trainer interview)

Workers with less experience in the mental health field also reported benefits, for instance;

*I found it massively beneficial to use it as a framework for my whole practice, learning what to do and how to communicate. Yeah, I think I took more from it than people who have already been in the role and already in mental health because it was all so fresh and new to me that I felt able to really take it on.* (Interview, Support Time and Recovery worker, Participant 15, SLaM)

Being prepared to try out techniques, tools and exercises, occasionally by suspending their scepticism, allowed clinicians to receive direct personalised feedback which challenged their assumptions about themselves and their service users. It also led to a few clinicians reporting a breakthrough with service users which they shared with colleagues, trainers and researchers.

*Primarily a willingness to participate, explore and ‘permission to play’ offered by the leadership present and also this themselves created the crucial element of success and why I think these sessions worked so well. The working contract included the commitment to openness and honesty on the part of the doctors and senior leads as well as the wider team to ‘try things out’ and not to be concerned about having to be ‘perfect’.* (CfR training reports, Team 4)

*It has actually encouraged me to put my assumptions aside, to think ‘is what we want always the best?’ I started to implement the methods and was pleasantly surprised at the fact that it was just like opening a door with a key with the patients. (Interview, Nurse, Participant 11, SLaM)*

### Effective training

#### Engagement strategies

On the whole, the teams responded very differently to the two types of externally provided training, with Coaching for Recovery training being better received. In terms of engaging the teams, a number of strategies appear to have been successful. The initial approach used to introduce and sell the training was crucial.

*The* [Coaching for Recovery ] *training day was very very different. I mean, the core thing is to say is ‘gosh guys you know so much already, let's just see if we can use all the knowledge and just look at different angles from it or fill in a few of the gaps here and there, I'll give you a few short cuts’. She got everyone onboard.* (Interview, Nurse, Participant 21, SLaM)

Despite being randomly assigned by an independent clinical trials unit to the intervention arm of the trial, some teams and senior managers, particularly in the less research-active trust, distrusted this allocation process. Responding to this observation, the research team met teams and senior managers to give more a detailed explanation of how and why random allocation is essential for increasing the scientific value of a trial, but this did not appear to convince everyone. As a team, we have since reflected that trial methodology needs to be explained more fully, at an earlier stage. Failure to do this led to participants reporting feeling affronted and insulted, believing it was an implied criticism of their existing practice and even that they were being singled out to receive remedial recovery training. In this climate, the validation of existing clinical skills and experience was essential in addressing these feelings, whilst at the same time positioning training as offering additional skills, techniques and ways of thinking about their practice.

#### Delivery style and content

Participants preferred the delivery style of the coaching training, which contained more skills-based, practical exercises and discussions around their own case material. Some commented that they did not like the theoretical teaching style the Personal Recovery training but did value the opportunity to have a facilitated discussion of the practical obstacles of recovery-oriented practice within their own teams.

*In doing role plays, rehearsing things together, it also surfaced I think aspects about people's assumptions about what is it that that we are doing as a core business and I think we were able to have a kind of conversation around that. People loved the coaching, even people can generally be a bit cynical or be kind of, ‘God, do we have to do this?’ People have said to me that she managed to talk kind of tangibly and practically about the basics of engaging someone who maybe doesn't want to be engaged at the surface.* (Interview, Team leader, Participant 16, SLaM)

#### Modelling recovery principles

Where the training itself was consistent with recovery principles, it was most effective. There were opportunities for trainer to model parallel processes regarding the use of strategies for engaging reluctant teams, the use of the strengths-based approach, mutual learning and using a coaching rather than a directive style of interaction.

*With this team I invited them to challenge their apparent tendency [to] down-play their own ‘competence and success’ and instead to own these as areas of expertise and achievement pointing out that if they cannot do this for their own achievements how would they be in a position to invite service users to do acknowledge their positive achievements – this appeared to provoke a real ‘aha’ moment for the team.* (CfR training report, Team 11).

The coaching compentency of ‘Contracting’ was used to good effect to engage participants by creating a collaborative working relationship and successful learning environment. Contracting with teams to agree flexible working arrangements gave staff permission to respond to urgent clinical matters and to feel that their concerns were being heard and responded to. As a parallel process, it also demonstrated the applicability of contracting as a tool for motivating and engaging reluctant service users.

## Discussion

This study is the first process evaluation of a recovery-oriented complex intervention nested in an RCT. It aimed to identify factors which promote or inhibit efforts to routinely embed complex interventions into existing mental healthcare practice. It produced three key findings which generalise to the implementation and evaluation of complex interventions.

First, this study highlighted the importance of targetting the transition from practitioner intent to implement to actual implementation behaviour. This was achieved by building in role-plays with colleagues, followed by small-scale, pilot experiences of using the intervention with service users. This exposed practitioners to direct, personalised feedback on the impact of the intervention and enabled negative attitudes and assumptions about likely consequences to be powerfully challenged. The personal recovery training to promoting recovery-oriented practice through knowledge acquisition and values-based training appeared to be less popular and effective. In an observational study of recovery-oriented training in state hospitals, Tsai and colleagues also found that specific/practical training had a greater increase in staff pro-recovery attitudes compared to general/inspirational training [22].

Second, consistent with other research [17], our study demonstrates the central importance of organisational commitment. Our study shows how staff evaluate organisational commitment using three markers: *resource allocation* (e.g. ensuring staff duties were covered to allow them to fully engage in training and team reflection sessions), organisational *Key Performance Indicator* metrics, and organisational *outcome measures*. Farkas and colleagues [5] have similarly reported that the implementation of recovery-oriented programmes has been hampered by focussing solely upon the collection of mandatory, routine outcome data on traditional clinical outcomes (e.g. symptomatology, relapse rates and employment) which may be incompatible with recovery outcomes (e.g. self-esteem, empowerment and well-being).

Third, for team-level interventions like this, we found that broader and unrelated organisational change processes greatly impacted upon staff action, directly via staff resourcing and indirectly, through implementation motivation and willingness. As these change processes will doubtless continue and resource allocation in health systems should be sensitive to this context. The fairest test of implementation might not be on an area-wide basis as in this study, but rather preferentially targeting teams that are at a mid-life stage of development, with low staff turnover, leadership capacity to frame involvement as an opportunity rather than a burden, and existing in-team ‘champions’ for the intervention. This points to the need for methodological extension of cluster RCTs, for example by including an organisational readiness to change measure as an inclusion criterion for selecting *both* organisations *and* individual teams, when evaluating team-level interventions within a RCT.

Benedetto [23] distinguished between “evolutionary” versus “revolutionary” implementation methods, based upon the anticipated degree of organisational or systems change necessary to achieve the desired improvement. The REFOCUS intervention could be classified as having used evolutionary implementation methods. It involved leadership-authorised, external teams and facilitators who created an intervention, assisted with implementation, but did not radically change job descriptions or staffing patterns [24]. In contrast, the Implementing Recovery though Organisational Change (ImROC) programme is using what could be termed revolutionary implementation methods, to enable organisations to assess, plan and evaluate their own recovery against ten indicators. These indicators include establishing Recovery Colleges to drive the programmes forward, transforming the workforce by employing peer support workers, and ensuring organisational commitment in creating a conducive ‘culture’ [25]. We have found that in preparatory, qualitative research conducted at trial sites, and in subsequent findings reported here, participants consistently identified implementation barriers and facilitators which can only be influenced at senior executive board level and beyond, hence the need for more restrictive inclusion criteria in future cluster RCTs.

### Strengths and limitations

This study focussed upon the perspectives of staff and trainers as part of an evaluation of a complex recovery intervention which was designed to enable staff to increase recovery support for service users who had a primary diagnosis of psychosis. The validity of this qualitative study was strengthened by the use of data triangulation, (sources of data came from staff, team leaders and trainers), methodological triangulation (use of in-depth interviews, focus groups and written reports), investigator triangulation (use of different investigators in the analysis process) and environmental triangulation (two contrasting research settings). These triangulation processes highlighted similarities and differences and enabled these to be examined to deepen the meaning in the data [21].

Some caution however, should be taken when considering the findings. The interview and focus group sample is purposive, with an inclusion criterion of interviewee's self-reported use of the intervention. We do not claim to represent the views and experiences of the entire population of staff working in intervention teams. There is also potentially a recall bias as the interview and focus groups were based on participant's recall of events over the 12 month period of the intervention. Recall bias and discrepancies are therefore likely to have occurred and present problems in terms of accuracy and reliability [26].

As all new programmes or interventions occur within a wider open system, they cannot be kept fully isolated from unanticipated events, policy changes, staff turnover, organisational targets and initatives, so identifying how these wider contextual organisational and environmental factors influence the uptake and success of an intervention is important. A limitation of this study is the failure to use a programme evaluation approach, such as proposed by Pawson and Tilley [27], to sufficiently link and examine the impact of these policy and organisational changes to the implementation of the intervention.

## Supporting Information

Figure S1 **Trainer's report guide.**
(DOCX)

Figure S2 **Interview schedule.**
(DOCX)

Figure S3 **Focus topic guide.**
(DOCX)
