# Supplementary material for: Implementing a Complex Intervention to Support Personal Recovery: A Qualitative Study Nested within a Cluster Randomised Controlled Trial
Source: PLoS One. 2014 May 29;9(5):e97091. doi: 10.1371/journal.pone.0097091 (PMC4038471; doi:10.1371/journal.pone.0097091)
Supplement: Figure S1 — Trainer's report guide. (DOCX) [file pone.0097091.s001.docx]

**Figure S1: Trainer’s report guide**

**Personal recovery and Coaching for recovery: Trainer’s report**

**2. Trainer’s notes and reflections**

*At the end of the Personal Recovery/Coaching for recovery training course (i.e. after the three sessions), please complete one report for each team.*

Trainer’s name:

Date of completion:

Report completed for (team name/borough/locality):

1. What were your impressions of the training overall?
2. What worked well and what didn’t work well?
3. How well was the training received?
   - By the team managers/leaders
   - By the psychiatrist
   - By the rest of the team
4. Were the team able/willing to apply the training to their practice? What helped and hindered this?
