# Supplementary material for: Implementing a Complex Intervention to Support Personal Recovery: A Qualitative Study Nested within a Cluster Randomised Controlled Trial
Source: PLoS One. 2014 May 29;9(5):e97091. doi: 10.1371/journal.pone.0097091 (PMC4038471; doi:10.1371/journal.pone.0097091)
Supplement: Figure S2 — Interview schedule. (DOCX) [file pone.0097091.s002.docx]

**Figure S2: Staff interview schedule**

**REFOCUS STAFF INTERVIEW SCHEDULE (Process evaluation)**

The interview will focus on experience of being in the intervention arm of the REFOCUS trial and implementing the REFOCUS intervention as set out in the REFOCUS manual and training. It will ask for specific practice examples of implementing the intervention components, how it has been implemented in routine clinical practice and how the intervention and implementation strategies could be developed and improved.

The interview will last up to one hour with an additional fifteen minutes to complete consent and respondent demographic data.

**Research objectives**

These semi-structured interviews explore the experiences of staff on implementing recovery orientated practice. There are four key research objectives: to explore,

- the understanding of recovery and the impact of personal recovery training on understanding
- the experience of implementing recovery intervention(s) as set out in the REFOCUS manual
- the barriers and facilitators to implementing recovery intervention(s) as set out in the REFOCUS manual
- how the REFOCUS intervention and implementation strategies could be developed and improved

**INTRODUCTION AND CONSENT**

- Aim: To introduce the research, clarify the content of the interview, explain confidentiality and gain consent.
- Introduce self and research. Trying to find out what part in the REFOCUS intervention has meant for you, in what way has the REFOCUS study intervention changed the way you work with examples. And what enabled this.
- Participation is voluntary and participant can withdraw at any time either before, during or after the interview
- Explain confidentiality assurances (confidential unless participant reports unsafe practice against code of conduct)
- Recording (to gain accurate record of discussion, allow interviewer to focus on what respondent is saying, only research team will hear it)
- Length (about an hour with breaks if needed)
- Nature of discussion (conversational in style with specific topics to be addressed)
- Place of interview (need for private space to conduct the interview)
- Reporting and data storage (no-one identified in final report, data stored securely under Data Protection legislation – can only be used for purpose collected by law, e.g. transcripts kept in locked cabinets, not shared with anyone outside research team.
- Address any questions
- Request written consent

**CURRENT CIRCUMSTANCES – SOCIODEMOGRAPHICS FORM**

- Aim: To gain background information about the respondent, to explore their staff role and to identify key characteristics of staff that are more likely to implement recovery.
- gender
- age
- education level
- team model of practice
- work role
- core profession
- grade
- length of time since qualification
- length of time in current post
- length of work experience in mental health services
- experience of mental illness
- use of mental health services
- experience of supporting a family member/friend with mental illness

**INTERVIEW QUESTIONS**

**Notes to interviewer:**

Remember to probe and get specific examples of implementing REFOCUS

If a participant misunderstands recovery or the intervention then you can correct / prompt

This is semi-structured and so not rigid

**[START RECORDING]**

**STAFF UNDERSTANDING AND EXPERIENCES OF REFOCUS intervention**

Aims:

To identify how the REFOCUS intervention has been understood, to identify staff experiences of implementing the REFOCUS intervention, illustrated by specific practice examples.

Opening questions

- Can you tell me a little bit about your role in the team?
- How have you found the REFOCUS intervention so far?

Section 1. Personal Recovery training

- Aim – to understand what it was like to have the personal recovery training
- **What was your experience of the personal recovery training?**
- Prompts:
- IF don’t remember: Run by rethink, with Sally and Vanessa or Laura (lived experience)
- How has the personal recovery training shaped how you view personal recovery?
- Have *attitudes* about recovery changed?
  - **Please can you give me an example as to how the recovery training has affected your practice?**
  - **How (else) would you like to use the recovery training in your clinical practice?**

Reflection sessions

- **How are you finding (did you find) the team reflection sessions?**
- **How has reflection supported you to implement the REFOCUS intervention?**
- **Have you been able to complete the supervision reflection form?**
  - Prompt: Show page 38 in manual
  - And how did this aid your supervision?
- **TEAM LEADERS ONLY: How are you finding the team leader reflection sessions?**

Coaching training

- **How has the coaching training altered how you work with service users?**
- **Please could you give me an example of how you use coaching skills:**
- Prompts
  - Contracting, Exquisite listening, Powerful questions, Challenge and confrontation, Goal setting, Feedback.
- **How many of your clients would you say you have you used coaching skills with, a rough percentage?**
  - Prompts
  - Have coaching skills become a part of your everyday practice?

Information session and manual

- **Did you attend the information session?**
- **What was the information session like?**
  - Prompts:
  - What else did you need to know about the REFOCUS intervention at the start?
- **Have you used the manual?**
  - Prompt: show manual
  - Which parts of the manual did you use?
  - Were they helpful?
- **How have you found the manual has affected your practice?**

Using the intervention

- - - **How do you explain or introduce the intervention to your service users?**

Prompt: What language do you use?

Is it the same language as in the manual or training?

- - - **How do you link the intervention into your everyday work?**

Prompt: Is there a better time to approach people?

Do you like to use it with other tools or assessents you use with service users?

Working practices: Values and treatment preferences

- **Can you give an example in the last 6 months of when you have been able to discuss values and treatment preferences with clients?**
- **How many clients have you discussed treatment values and preferences with?**
- **Did you use the VTP guide? How did you find using the VTP guide?**
  - Prompt: manual page 28
- **Did you use any other approaches to talking to people about their values and treatment preferences?**
  - Prompt: manual pages 14 & 15

Working practices: Assessing Strengths

- **Please can you give me an example in the last 6 months of when you have assessed a clients strengths?**
- **How many of your clients have you assessed strengths with?**
- **How did you find the strengths worksheet?**
  - Prompt: manual pages 32 - 36

Working practices: Goals

- **Has it been possible to support goal striving and the identification of personally valued goals? How and with what effect?**
- **Please can you give me an example?**
- **Has it been possible to record all these things (strengths, vtp, goals) on the clinical information system?**

Working relationship

- **Has the REFOCUS intervention changed your relationships with clients and how? [own values, coaching skills, raised expectations of service users]**
- **Do you feel the language you use with your clients has changed in the last 6 months?**
- **Has the balance of power changed? If so can you give an example of a shift in the power balance since starting the REFOCUS intervention?**
- **Has the way you view the duty of care and how it fits with recovery principles changed?**
  - Example of dealing with risk in a recovery orientated way
- Partnership Project
- **Has your team bid for the Partnership project money?**
- **If yes, please describe the project / activity the money was for.**
- **How have you found being involved in a partnership project?**
- **If no, Why do you think your team haven’t applied? What barriers have you faced?**
- General comments
- **Is there anything else you would like to say about how you found implementing the REFOCUS intervention?**  Any barriers or facilitators [general or specific to each working practice [values and treatment preferences, strengths, goals], recovery-promoting relationship]?
- **Which parts of the REFOCUS intervention did you think were feasible?**
- **Would you change the REFOCUS intervention either the content or the implementation strategy? If so, how?**
  - Drop something?
  - Alter something?
- **What changes do you think service users have noticed from your use of the REFOCUS intervention?**
- **Is there anything else you would like to say about the REFOCUS intervention?**
- End of interview. Thank respondent and close interview.
