# Supplementary material for: Implementing a Complex Intervention to Support Personal Recovery: A Qualitative Study Nested within a Cluster Randomised Controlled Trial
Source: PLoS One. 2014 May 29;9(5):e97091. doi: 10.1371/journal.pone.0097091 (PMC4038471; doi:10.1371/journal.pone.0097091)
Supplement: Figure S3 — Focus topic guide. (DOCX) [file pone.0097091.s003.docx]

**Figure S3: Focus group topic guide**

**REFOCUS trial focus group topic guide – staff**

**Research objectives:**

- To explore variation across intervention teams (recruit both high and low implementing teams)
- To explore the team-level experience of implementing recovery intervention(s) as set out in the REFOCUS manual
- To explore the barriers and facilitators to implementing recovery intervention(s) as set out in the REFOCUS manual

1. Introduction and consent

*“We are interested in gathering feedback on your experiences of implementing the REFOCUS intervention as a team, and how you’ve all supported recovery in your practice.”*

**Focus group questions**

Section 1: TEAM UNDERSTANDING AND EXPERIENCES OF RECOVERY [25 minutes]

1. **When I say the phrase “Personal Recovery” what thoughts immediately spring to mind?**

AIMS:

To identify whether teams frame their practice in terms of recovery

To identify how they relate REFOCUS to personal recovery, and whether the intervention has changed their understanding of the recovery model

Prompts / Continuation Questions:

- What are the team’s priorities and goals for practice?
- What is it that happens in this team that supports the recovery of your service users?
- How would you say the REFOCUS Manual fits in with the personal recovery approach?
- How has your understanding of recovery been enhanced since being involved in the REFOCUS study?

1. **As a team, how have you found implementing the REFOCUS Manual with your service users?**

AIMS:

To identify team experiences of implementing the REFOCUS intervention

Prompts / Continuation Questions:

- What examples do you have of using your experiences in REFOCUS to support service users?
- What changes have you noticed in the team’s practice since taking part in the REFOCUS study?

Section 2: BARRIERS AND FACILITATORS TO IMPLEMENTING RECOVERY [25 minutes]

1. **Can you tell me what it’s been like for your team to try and implement the REFOCUS intervention?**

AIMS:

To explore the team’s level of implementation, the circumstances surrounding implementation, and how/why this occurred

To explore team experiences of successful or unsuccessful implementation

Prompts / Continuation Questions:

- Could you discuss what you felt were the most helpful parts of the REFOCUS intervention?
- Could you discuss what you felt were the least helpful parts of the REFOCUS intervention?

The REFOCUS Strategies:

Information Sharing Sessions

Personal Recovery Training

Coaching Training

Team Reflection

Individual Reflection

Partnership Project Event

- How has the ethos of the team affected your implementation of the REFOCUS intervention?
- How has the Trust organisation affected your implementation of the REFOCUS intervention?

*If implementation has been perceived as successful:*

1. **What is it about your team that enables you to successfully support recovery?**

Prompts / Continuation Questions:

- Is there a shared understanding of recovery within the team?
- In what ways has the REFOCUS intervention become part of your team’s approach to care?

*If implementation has been perceived as low:*

1. **What would further support your team to implement the REFOCUS Manual?**

Prompts / Continuation Questions:

- What resources would better equip the team to implement the REFOCUS working practices?
- What would you say is the ideal environment to implement the REFOCUS Manual in?

The REFOCUS working practices:

Understanding values and treatments preferences

Assessing strengths

Supporting goal-striving

Section 3: CLOSE THE FOCUS GROUP [10 minutes]

1. **This focus group was designed to help us understand the extent to which the REFOCUS intervention has been implemented, and what has helped or hindered your team in doing this. Are there any other important points that you would like to discuss before we close the discussion?**

End of focus group. Thank all participants, and close the discussion.
